# Generative AI in peer review process for occupational health

**DOI:** 10.1093/occmed/kqaf051

**Published:** 2025-07-02

**Authors:** G H Lim, M L Tan, V C W Hoe, D Koh

**Affiliations:** Saw Swee Hock School of Public Health, National University of Singapore, Singapore 117549; Department of Occupational Medicine, Sengkang General Hospital, Singapore 544886; Centre for Epidemiology and Evidence-Based Practice, Department of Social and Preventive Medicine, Faculty of Medicine, Universiti Malaya, 50603 Kuala Lumpur, Malaysia; Saw Swee Hock School of Public Health, National University of Singapore, Singapore 117549; University of Occupational and Environmental Health, Kitakyushu, Fukuoka Prefecture 807-0804, Japan

## Abstract

**Background:**

Generative Artificial Intelligence (AI) tools in academic writing can augment and speed up the proofing process by improving sections of the manuscript. This was the first known instance where the effectiveness and efficiency of Generative AI were quantified.

**Aims:**

To determine the effectiveness and efficiency of these tools in providing feedback and recommendations to the first drafts of eight published occupational health papers.

**Methods:**

To assess effectiveness, manuscripts were reviewed by Microsoft Copilot, ChatGPT (GPT-3.5), Google Gemini 1.0 and five human reviewers in February 2024. Anonymized reviews were scored by two expert panel members using a self-developed structured scoring system. The quality of feedback was rated on its relevance, completeness, accuracy and ability to identify errors and provide constructive feedback. The quality of recommendations was rated on relevance, completeness and accuracy. Efficiency was assessed via the time taken to complete each review. The mean, standard deviation (SD) and level of significance of any differences among the parameters were obtained.

**Results:**

Generative AI tools were significantly more effective (3.44, SD 0.77, *P* < 0.001) than human reviewers in providing feedback, while human reviewers performed significantly better (3.36, SD 0.71, *P* < 0.01) in providing recommendations. Generative AI tools were significantly more time-efficient and had the advantage of being always available. However, time/effort was required to verify the output for fictitious content.

**Conclusions:**

The utilization of Generative AI would improve the speed and accuracy of improving the manuscript prior to publication, leading to greater efficiencies in the dissemination of knowledge to the occupational health community.

## Teaser

This study quantified the effectiveness and efficiency of Generative Artificial Intelligence (AI) tools in providing feedback to the first drafts of published occupational health papers. Generative AI tools were more effective in providing feedback, while human reviewers performed better in providing recommendations. Generative AI tools were significantly more time-efficient but not task efficient. The utilization of Generative AI tools would lead to greater efficiencies in the dissemination of knowledge to the occupational health community.

**Key learning points**

**What is already known on this subject:**

- Generative Artificial Intelligence (AI) tools in the field of academic writing can augment and speed up the proofing process by improving sections of the manuscript.
- This novel study quantified the effectiveness and efficiency of Generative AI tools in providing feedback to the first drafts of published occupational health papers.

**What this study adds:**

- The effectiveness of Generative AI tools and human reviewers was graded based on the quality of feedback and recommendations, while efficiency was assessed via the time taken to complete each review.
- Generative AI tools provided more effective feedback, while human reviewers provided better recommendations. Generative AI tools were significantly more time-efficient.

**What impact this may have on practice or policy:**

- The utilization of Generative AI tools would lead to greater efficiencies in the dissemination of knowledge to the occupational health community.
- Users would need to be familiar with the strengths and limitations of each Generative AI tool and be aware that factual errors might occur.

## INTRODUCTION

In recent years, Generative Artificial Intelligence (AI) tools had been adopted in many aspects of personal and professional life. Many tasks associated with the preparation of a manuscript could be performed by these tools. These include editing, summarizing, idea generation, data analysis, evaluation and coming out with recommendations [1–4].

The ability to improve writing style and generate new ideas, instant availability and improved efficiency make these tools essential to augment researcher productivity [3, 5]. The utilization of these tools to augment researchers would improve the speed and accuracy of improving the manuscript prior to publication, leading to greater efficiencies in the dissemination of knowledge to the occupational health community.

While efficient and always available, these tools were not without limitations. The original models required users to verify generated content through fact-checking and cross-referencing [6]. In one study, authors noted that none of the publications provided as a reference appeared in Google Scholar, suggesting that Generative AI tools might fabricate evidence [7].

Generative AI tools were also prohibited for certain aspects of manuscript development [8]. These include creation and alteration of images, and crediting of such tools as an author [9]. Many journals also required documentation on usage of these tools, and limited usage to improving readability and language [10].

Other concerns included worries of deterioration in critical thinking [11] and writing skills [2]. Given the widespread availability and capability of such tools to prepare a significant portion of a manuscript, there was also potential for irrelevant manuscript submission, whose sole purpose was to generate a journal publication [4].

While much was known on the effectiveness and applications of these Generative AI tools, till date, the authors were not aware of any articles quantifying the effectiveness and efficiencies of these tools.

Therefore, we aimed to determine the effectiveness of Generative AI tools in providing feedback and recommendations to the first drafts of eight published occupational health research papers. The secondary aim of the study was to compare the efficiency of Generative AI tools versus human reviewers.

## METHODS

**Figure 1.**
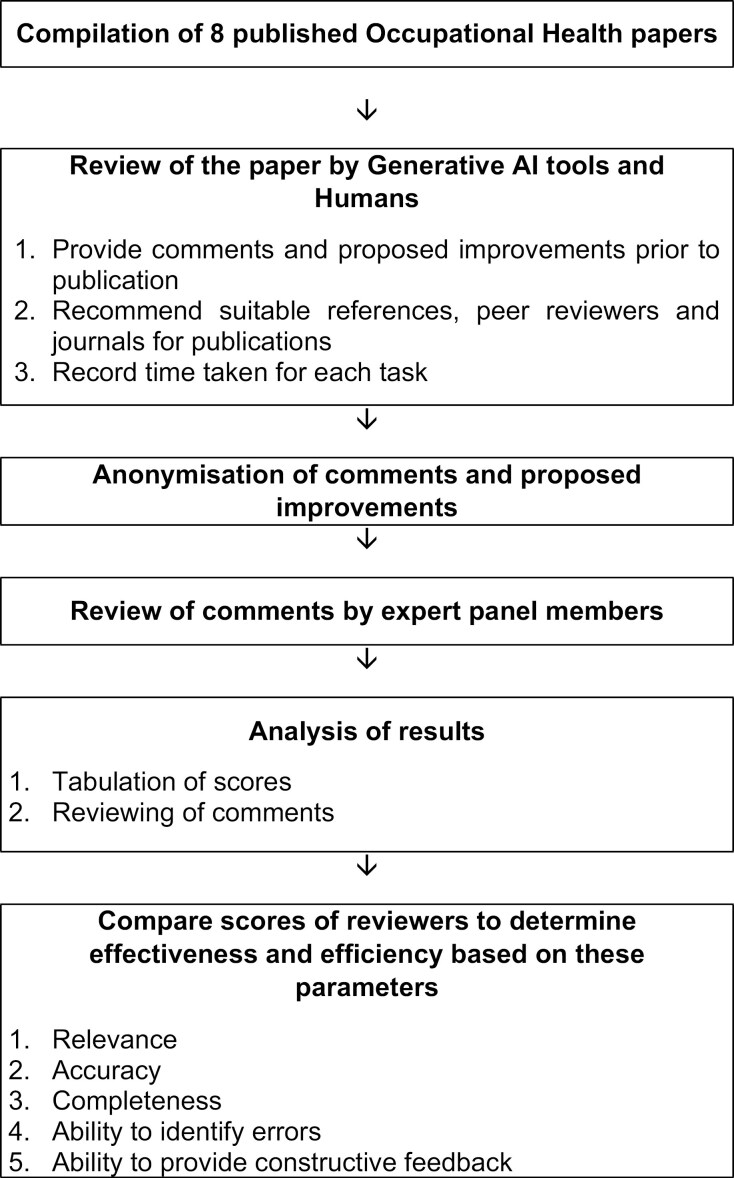
Study workflow.

The study was performed in two phases. In Phase 1, reviewers were asked to provide their inputs on eight first drafts of published occupational health manuscripts. In Phase 2, an expert panel was asked to grade these inputs using a self-developed scoring system. The overall study workflow is detailed in Figure 1.

For Phase 1, the eight reviewers included three Generative AI tools and five human participants. Generative AI tools were selected if they were open access (without a need for paid subscription) and used the latest language model (at least GPT-3.5 or equivalent). These included Microsoft Copilot [12], ChatGPT (GPT-3.5) [13] and Google Gemini 1.0 [14], which were accessed in February 2024 Table 1 (available as Supplementary data at *Occupational Medicine* Online) . Human participants were selected if they were Occupational Medicine residents who were attending or achieved Master of Public Health and had authored at least one peer-reviewed published paper.

For Phase 2, the expert panel was made up of two members. The inclusion criteria included being a senior Occupational Medicine consultant who had published at least 50 peer-reviewed publications on occupational health and had experience as a member in an editorial board of at least two international peer-reviewed journals.

The study was opened to all Occupational Medicine residents who met the inclusion criteria. Study details were shared via an official residency mailing list. Announcements were also made during weekly residency teaching sessions. Interested participants registered by providing their full name and email address. They were then briefed on the study protocol using the Participant Information Sheet. Informed consent was obtained using the Participant Consent Form, with both physical and digital copies archived Appendix A (available as Supplementary data at *Occupational Medicine* Online).

As there were challenges in obtaining the first draft of published papers, the convenience sampling method was chosen, where existing and recently exited Occupational Medicine residents from the National Preventive Medicine Residency, Singapore were approached for the first draft of their published papers. A total of eight research papers were eventually obtained in this manner for review. These included five research articles, one review, one commentary and one case report Table 2 (available as Supplementary data at *Occupational Medicine* Online).

In Phase 1, three Generative AI tools, namely ‘Microsoft Copilot’, ‘ChatGPT (GPT-3.5)’ and ‘Google Gemini 1.0’, as well as five human reviewers, were asked to provide their inputs on six different sections of each research paper, namely ‘Abstract’, ‘Introduction’, ‘Materials and Methods’, ‘Results’, ‘Discussions’ and ‘Conclusions’ Appendix B (available as Supplementary data at *Occupational Medicine* Online). Due to variations in publication types and journal requirements, reviewers were specifically informed to grade missing sections as ‘non-applicable’.

A standardized set of prompts on how to improve the paper were posed to both Generative AI tools and human reviewers Table 3 (available as Supplementary data at *Occupational Medicine* Online). In addition, reviewers were asked to suggest relevant references and provide recommendations for peer reviewers and journals for publication. Noting the variations in the data input requirements for the three Generative AI tools, data input methods were slightly modified to obtain the same output. For example, each section was broken into different parts for Microsoft Copilot due to its word limit per input Table 4 (available as Supplementary data at *Occupational Medicine* Online). A total of 64 submissions were generated by the eight reviewers (three Generative AI tools and five human reviewers), who reviewed eight publications each. These were anonymized before being reviewed by the expert panel.

In Phase 2, all 64 submissions were scored by the expert panel using a self-developed structured scoring system to assess the effectiveness of the review. The quality of feedback was rated on its relevance, completeness, accuracy and ability to identify errors and provide constructive feedback. The quality of recommendations was rated on its relevance, completeness and accuracy.

The panel graded each submission on the quality of feedback using five parameters, namely ‘Relevance of feedback’, ‘Completeness of feedback’, ‘Accuracy of feedback’, ‘Ability to identify errors’ and ‘Ability to provide constructive feedback’. Similarly, the ability to provide recommendations was graded using three parameters, namely, ‘Relevance of recommendation’, ‘Completeness of recommendation’ and ‘Accuracy of recommendation’. Each factor was scored using a 5-point Likert scale, where 1 is the least effective and 5 is the most effective Appendix C (available as Supplementary data at *Occupational Medicine* Online). To minimize variations in scoring, a detailed scoring criteria were shared with the expert panel members Appendix D (available as Supplementary data at *Occupational Medicine* Online).

To assess effectiveness, each expert panel member reviewed and scored 64 submissions using this criteria. The mean score for each parameter was then tabulated to determine the effectiveness of each reviewer in the domain of providing feedback and domain of providing recommendations.

To assess efficiency, all eight reviewers recorded the time spent on each section during the review process. The overall mean time spent on each paper was obtained. At the macro level, the number of days taken for the reviewers to process and submit all manuscripts was recorded. The number of days taken for the expert panel members to grade the submission was also recorded. These results were analysed using the statistical software ‘R Studio’ 2023.12.1+402 to obtain the mean, standard deviation (SD) and level of significance of any differences among the parameters. Ethical approval was obtained from Saw Swee Hock School of Public Health Departmental Ethics Review Committee (SSHSPH-228).

## RESULTS

In terms of overall effectiveness of feedback, Generative AI tools performed significantly better with a mean score of 3.44 (SD 0.77) out of 5.00 compared to human reviewers at 2.33 (SD 0.68), with *P* <0.001. Compared to human reviewers, feedback by the Generative AI tools was assessed to be significantly more relevant, more complete, more accurate, better at identifying errors and more constructive with a mean score of 3.50 (SD 0.76), 3.54 (SD 0.72), 3.45 (SD 0.79), 3.22 (SD 1.00) and 3.52 (SD 0.72), with *P* <0.001 for all sections.

Meanwhile, human reviewers performed significantly better on providing recommendations, with a mean score of 3.36 (SD 0.71) out of 5.00 compared to Generative AI tools at 2.85 (SD 0.92), with *P* <0.01. Compared to Generative AI tools, recommendations by human reviewers were assessed to be significantly more relevant, more complete and more accurate with a mean score of 3.38 (SD 0.75), 3.35 (SD 0.69) and 3.35 (SD 0.75), with *P* <0.05 for all sections.

In terms of overall efficiency, Generative AI tools performed significantly better with a mean time of 11.08 minutes (SD 3.57) compared to human reviewers at 45.15 minutes (SD 4.79), with *P* <0.001. On average, human reviewers took 34.4 days to review and submit the papers. Meanwhile the author took an average of 1.5 days to review the papers with the help of the Generative AI tools. The expert panel members took an average of 30.5 days to grade the submissions. The overall effectiveness and efficiency of Generative AI tools compared to human reviewers are summarized in Table 1.

**Table 1.** Effectiveness and efficiency of Generative AI tools compared to human reviewers

Effectiveness in providing feedback and recommendation				
Characteristic	Overall, *N* = 128	AI, *N* = 48	Humans, *N* = 80	*P*-value
Feedback^a^				
Relevance	2.75 (0.92)	3.50 (0.76)	2.30 (0.68)	<0.001
Completeness	2.70 (0.95)	3.54 (0.72)	2.19 (0.67)	<0.001
Accuracy	2.70 (0.92)	3.45 (0.79)	2.25 (0.68)	<0.001
Error identification	2.55 (1.00)	3.22 (1.00)	2.14 (0.77)	<0.001
Constructive	2.73 (0.92)	3.52 (0.72)	2.25 (0.67)	<0.001
Overall	2.68 (0.92)	3.44 (0.77)	2.23 (0.68)	<0.001
Recommendation^a^				
Relevant	3.18 (0.85)	2.83 (0.91)	3.38 (0.75)	0.003
Complete	3.17 (0.83)	2.88 (0.95)	3.35 (0.69)	0.009
Accurate	3.16 (0.85)	2.84 (0.92)	3.35 (0.75)	0.013
Overall	3.17 (0.83)	2.85 (0.92)	3.36 (0.71)	0.008

Efficiency in reviewing				
Paper	Overall, *N* = 64	AI, *N* = 24	Humans, *N* = 40	*P*-value
1	27.94	7.16	40.40	<0.001
2	36.56	11.50	51.60	<0.001
3	31.18	9.50	44.20	<0.001
4	34.37	9.33	49.40	<0.001
5	37.31	15.50	50.40	<0.001
6	26.56	6.50	38.60	<0.001
7	32.37	13.33	43.80	<0.001
8	32.68	15.83	42.80	<0.001
Overall^b^	28.12 min (18.06 min)	11.08 min (3.57 min)	45.15 min (4.79 min)	<0.001

^a^Mean score (SD) out of maximum score of 5.00.

^b^Mean time (SD).

Comparing the three Generative AI tools, there were no significant differences in the overall effectiveness of providing feedback. Comparing the overall effectiveness in providing recommendations, ChatGPT (GPT-3.5) performed significantly better with a score of 3.21 (SD 0.43) out of 5.00 compared to Microsoft Copilot at 3.13 (SD 0.89) and Google Gemini 1.0 at 2.22 (SD 1.01), with *P* <0.01. In terms of overall efficiency, ChatGPT (GPT-3.5) performed significantly better with a mean time of 4.13 minutes (SD 0.44) compared to Google Gemini 1.0 at 5.00 minutes (SD 1.07) and Microsoft Copilot at 24.13 (SD 9.98), with *P* <0.001. The overall effectiveness and efficiency of Generative AI tools are summarized in Table 2.

**Table 2.** Effectiveness and efficiency between Generative AI tools

Effectiveness in providing feedback and recommendation					
Characteristic	Overall, *N* = 48	Microsoft Copilot, *N* = 16	Google Gemini 1.0, *N* = 16	ChatGPT (GPT-3.5), *N* = 16	*P*-value
Feedback^a^					
Relevance	3.50 (0.76)	3.31 (0.89)	3.54 (0.74)	3.64 (0.64)	ns
Completeness	3.54 (0.72)	3.43 (0.78)	3.54 (0.77)	3.66 (0.62)	ns
Accuracy	3.45 (0.79)	3.25 (0.87)	3.51 (0.76)	3.58 (0.75)	ns
Error identification	3.22 (1.00)	3.10 (1.05)	3.34 (0.85)	3.20 (1.14)	ns
Constructive	3.52 (0.72)	3.41 (0.73)	3.52 (0.80)	3.64 (0.67)	ns
Overall	3.44 (0.77)	3.30 (0.85)	3.49 (0.76)	3.54 (0.72)	ns
Recommendation^a^					
Relevant	2.83 (0.91)	3.08 (0.86)	2.21 (1.01)	3.21 (0.44)	<0.01
Complete	2.88 (0.95)	3.23 (0.96)	2.23 (1.03)	3.19 (0.42)	<0.01
Accurate	2.84 (0.92)	3.08 (0.90)	2.21 (1.01)	3.23 (0.43)	<0.01
Overall	2.85 (0.92)	3.13 (0.89)	2.22 (1.01)	3.21 (0.43)	<0.01

Efficiency in reviewing					
Paper	Overall, *N* = 24	Microsoft Copilot, *N* = 8	Google Gemini 1.0, *N* = 8	ChatGPT (GPT-3.5), *N* = 8	*P*-value
1	7.2	14.0	4.0	3.5	<0.001
2	11.5	23.0	7.0	4.5	<0.001
3	9.5	19.0	5.0	4.5	<0.001
4	9.3	19.0	5.0	4.0	<0.001
5	15.5	37.0	5.0	4.5	<0.001
6	6.5	12.0	4.0	3.5	<0.001
7	13.3	32.0	4.0	4.0	<0.001
8	15.8	37.0	6.0	4.5	<0.001
Overall^b^	11.08 min (10.94 min)	24.13 min (9.98 min)	5.00 min (1.07 min)	4.13 min (0.44 min)	<0.001

ns, non-significant.

^a^Mean score (SD) out of maximum score of 5.00.

^b^Mean time (SD).

Compared to human reviewers, responses by Generative AI tools provided more detailed feedback in addition to automatically generated revised versions of each section Table 5 (available as Supplementary data at *Occupational Medicine* Online). Microsoft Copilot and Google Gemini 1.0 also provided up-to-date feedback and recommendations as they were able to perform real-time searches. See Table 3 for a summary of the capabilities of Generative AI tools as of February 2024.

**Table 3.** Summary of capabilities of Generative AI tools

	Microsoft Copilot	ChatGPT (GPT-3.5)	Google Gemini 1.0
Capabilities^a^	Information retrieval Language translation Creative writing Code generation Image understanding Error identification Conversation	Information retrieval Language translation Creative writing Code generation Image understanding Error identification Conversation	Information retrieval Language translation (via Google translate) Creative writing Code generation Image search (via Google search) Error identification Conversation
Input	~7500 characters per interaction	4096 characters per interaction	No limit
Output	Accurate, consistent and updated	Accurate	Accurate, consistent and updated
Output speed	Slow. Text appears letter by letter until the output is completed.	Fast	Fast
Output length	Comprehensive but lengthy	Comprehensive but lengthy	Comprehensive but lengthy
Limitations in providing recommendations	No limitations	Limited by algorithm against providing recommendations on Peer Reviewers.	Limited by algorithm against providing recommendations on References, Peer Reviewers and Journal.
Accuracy	May get information wrong sometimes	Hallucinations—fabricates a plausible answer when it does not know	May get information wrong sometimes
Possible uses	Coding, research	Conversation	Informative answers

^a^As of February 2024.

Limitations of Generative AI tools included lengthy responses, word limit for chat inputs and restrictions on output content due to developer policies. Generative AI tools also created plausible sounding fictitious content, a situation more commonly known as ‘hallucinations’ [2]. See Table 4 for a summary on the frequency and types of limitations in the responses by the Generative AI tools.

**Table 4.** Frequency and types of limitations in Generative AI tools responses

Section	Types of limitations in Generative AI tool responses	Microsoft Copilot, *N* = 8	Google Gemini 1.0, *N* = 8	ChatGPT (GPT-3.5), N = 8
References	Avoids answering inputs directly	3	0	0
	Unable to provide recommendations	0	1	0
	Not allowed by algorithm	0	1	0
Reviewers	Avoids answering inputs directly	1	0	0
	Unable to provide recommendations	1	1	4
	Not allowed by algorithm	0	7	0
	Hallucinations	0	0	3
Journals for Publication	Avoids answering inputs directly	0	0	0
	Unable to provide recommendations	0	3	0
	Not allowed by algorithm	0	2	0

Microsoft Copilot was limited in providing recommendations for the ‘References’ and ‘Reviewers’ sections. In the output for the ‘References’ section, there were three out of eight instances where it avoided answering the inputs directly. For example, Microsoft Copilot stated that ‘Depending on the citation style and the type of source you are using, you may need different information to cite your sources correctly. Here are some general guidelines and examples for common source types’ [12]. Meanwhile, in the output for the ‘Reviewers’ section, there was one out of eight instances where it was unable to provide recommendations. For example, Microsoft Copilot stated that “I’m sorry, but I cannot recommend specific peer reviewers for this research paper’. [12].

Google Gemini 1.0 was limited by its algorithm in providing recommendations for the ‘References’, ‘Reviewers’ and ‘Journals’ sections. In the output for the ‘References’ section, there was one out of eight instances where Google Gemini 1.0 was not allowed by algorithm to provide recommendations. For example, it stated that ‘Unfortunately, I cannot directly cite specific references for your paper as ethical guidelines prevent me from generating content that could be construed as academic work’ [14]. Meanwhile, in the output for the ‘Reviewers’ section, there were seven out of eight instances where Google Gemini 1.0 was not allowed by algorithm to provide recommendations. For example, it stated that ‘Unfortunately, I cannot directly recommend specific peer reviewers for your research paper due to limitations in accessing personal information and potential conflicts of interest’ [14]. Similarly, in the output for the ‘Journals’ section, there were two out of eight instances where Google Gemini 1.0 was not allowed by algorithm to provide recommendations. For example, it stated that ‘Unfortunately, I cannot directly recommend specific journals for your publication as it could be construed as academic advice’ [14].

ChatGPT (GPT-3.5) provided recommendations for ‘References’ and ‘Journals’ section but was limited by its algorithm for providing recommendations for the ‘Reviewers’ section. In the output for the ‘Reviewers’ section, there were four out of eight instances where ChatGPT (GPT-3.5) was unable to provide recommendations. For example, it states that ‘As an AI developed by OpenAI, I don’t have access to specific databases or information about individuals’ expertise’ [13] but tries to be helpful to provide advice on how the author can proceed to obtain such information. There were also three out of eight occasions where ChatGPT (GPT-3.5) ‘hallucinates’ and described a non-existent reviewer. For example, it recommended ‘Dr. Sarah Smith, Affiliation: Department of Aerospace Engineering, University of Aviation, Expertise: Dr. Smith’s research focuses on human factors in aviation, including fatigue management and crew resource management. She has published extensively in peer-reviewed journals on topics related to aviation safety and performance’ [13]. See Table 5 for more examples of responses provided by Generative AI Tools Table 6 (available as Supplementary data at *Occupational Medicine* Online).

**Table 5.** Example of responses provided by Generative AI tools

Would you be able to provide some recommendations for references?	
Microsoft Copilot	*Avoids answering input directly*: ‘Depending on the citation style that you are using, there are different sources that can guide you on how to cite references for a research paper. Here are some examples’.
Google Gemini 1.0	*Unable to provide recommendation*: ‘I’d be happy to help you find some relevant references for your research paper! However, to provide the most accurate and helpful recommendations, I’ll need some more information about your specific research focus and the fumigants you’re discussing’. *Not allowed by algorithm to provide recommendation*: ‘Unfortunately, I cannot directly cite specific references for your paper as ethical guidelines prevent me from generating content that could be construed as academic work. However, I can offer some suggestions and best practices for finding relevant references to cite based on your research topic’.
ChatGPT (GPT-3.5)	Provided recommendations for all papers

Would you be able to recommend two peer reviewers who might be familiar in this field?	
Microsoft Copilot	*Unable to provide recommendation*: ‘I’m sorry, but I cannot recommend specific peer reviewers for your research paper on fumigation and occupational health. However, I can help you with finding some sources that might be relevant and useful for your paper. You can use these sources to identify potential peer reviewers who have expertise and experience in this field’. *Avoids answering input directly*: ‘One way to find peer reviewers is to search for relevant and recent publications in your field, and look at the authors, editors, and reviewers of those papers’.
Google Gemini 1.0	*Not allowed by algorithm to provide recommendation*: ‘Unfortunately, I cannot directly recommend specific peer reviewers for your study due to limitations regarding personal information and potential conflicts of interest. However, I can suggest some approaches to help you identify suitable reviewers’
ChatGPT (GPT-3.5)	*Hallucinations*: ‘Dr. Sarah Smith, Affiliation: Department of Aerospace Engineering, University of Aviation, Expertise: Dr. Smith’s research focuses on human factors in aviation, including fatigue management and crew resource management. She has published extensively in peer-reviewed journals on topics related to aviation safety and performance’. *Unable to provide recommendation*: ‘As an AI developed by OpenAI, I don’t have access to specific databases or information about individuals’ expertise. However, I can suggest a couple of general strategies to identify potential peer reviewers for your paper’

Would you be able to recommend two suitable journals for this publication?	
Microsoft Copilot	Provided recommendations for all papers
Google Gemini 1.0	*Unable to provide recommendation*: ‘While I can’t definitively recommend two specific journals without knowing more about your research, I can definitely guide you towards suitable choices based on the information you’ve provided so far’. *Not allowed by algorithm to provide recommendation*: ‘Unfortunately, I cannot directly recommend specific journals for your publication as it could be construed as academic advice. However, I can provide you with some factors to consider and resources to help you identify suitable journals for your research on undergraduate OM education’.
ChatGPT (GPT-3.5)	Provided recommendations for all papers

## DISCUSSION

We found that compared to human reviewers, Generative AI tools were significantly more effective in the domain of providing quality feedback during the simulated review process. Generative AI tools provided more relevant, complete, accurate and constructive feedback. They were better in error detection and had the added advantage of producing a revised version of the input.

Meanwhile, we found that human reviewers performed significantly better than Generative AI tools in the domain of providing quality recommendations. Human reviewers provided more relevant, complete and accurate recommendations. This was perhaps due to the limitations imposed by the Generative AI tool developers. For example, some Generative AI tools were not permitted to generate a list of relevant references and suggestions for peer reviewers due to ethical considerations.

With the current large language models (LLMs), there were concerns regarding the risk of Generative AI tools creating fictitious content—more commonly known as ‘hallucinations’ [2]. While useful in creative writing and in the idea generation phase (what the LLMs were designed for), this would be dangerous if fictitious references were created to support key ideas proposed in any research paper. It would be akin to a researcher fabricating results and thus fundamentally challenge the evidence-based approach of any scientific paper.

While some hallucinations could be easily detected, others were subtle and required careful verification. In this study, several instances of easily detected Generative AI hallucination were encountered, where the names of potential peer reviewers suggested would clearly trigger alarm bells—‘Sarah Smith’ and ‘John Doe’. This phenomena were well documented in another publication where all the references generated were found to be fictitious after verification with online scientific databases [7].

At the point of writing, it was noted that many journals prohibit the use of Generative AI tool for manuscript development [8], and required documentation should such tools be used [10]. This was probably in response to the fear of false data generation, which would compromise research integrity. It would be very likely that such risks would be lowered with improved LLMs in the future.

With the widespread use of Generative AI tools in many other domains, new frameworks would likely be developed to enable productive use of Generative AI tools to augment academic writing. These tools would then be no different from current statistical tools used for reducing computational workload during analysis of large datasets.

Our results, as expected, showed that Generative AI tools were significantly more time-efficient at reviewing the papers. In terms of availability, Generative AI tools also had the significant advantage of being always available. This was experienced during this study, when data collection was impeded because of work commitments and major public holidays in December, January and February.

As with all tools, the correct usage would generally improve time efficiency. However, time efficiency would not always equate to task efficiency. The misapplication of a Generative AI tool (e.g. using the current LLMs to generate references when there was a known risk of hallucinations) would inevitably result in more time/effort required to clean up the manuscript prior to submission.

An astute researcher would thus need to be familiar with the strengths and limitations of each Generative AI tool to yield maximum efficiency in the creation of publications for the peer review process. Among the three Generative AI tools examined in this study, each demonstrated aptitude for different sections. Researchers should consider the approach of using different Generative AI tools for different domains.

One key strength of this study was the 100% response rate from reviewers and expert panel members. The study design also replicated the workflow of the proofing process where the first draft of the occupational health paper was submitted to peers/mentors for initial comments before submission to the desired journal for review.

One limitation in the study design was the intentional use of free access Generative AI tools, which might not be representative of the Generative AI tools available in the market. The paid versions customized for research writing in the market had lesser limitations and were able to process graphics and tables. Nevertheless, this approach was chosen as free access Generative AI tools were assessed to be more accessible to researchers, and thus more representative of actual usage patterns.

In addition, the human reviewers were generally junior in their research career (residents with experience in at least one peer-reviewed publication). This could result in an entry-standard in the review quality compared to more experienced reviewers. We also acknowledge the small sample size of eight occupational health papers due to challenges in obtaining the first draft of published papers.

With the rapid advancement of Generative AI tools, development of customizable AI assistants trained on specific datasets of peer-reviewed articles and academic writing style of the journal is now possible [15]. This would enable more accurate and contextually relevant feedback. The use of such customizable AI assistants could streamline the peer review process, enhance the quality of academic writing and offer more reliable recommendations for research improvements.

Knowing the strengths and limitations of the Generative AI tools would permit utilization of these adjuncts to enhance the field of academic writing. The appropriate application of Generative AI tools to augment researchers during the generation of the first draft would lead to substantial efficiencies in publishing important occupational health knowledge.

## Supplementary Material

kqaf051_suppl_Supplementary_Appendixs

## References

[1] Salvagno M , Taccone FS, Gerli AG. Can artificial intelligence help for scientific writing? Crit Care 2023;27:75. 36841840 10.1186/s13054-023-04380-2 PMC9960412

[2] Lin Z. Techniques for supercharging academic writing with generative AI. Nat Biomed Eng 2025;9:426–431. 38499642 10.1038/s41551-024-01185-8

[3] Anderson LB , Kanneganti D, Mary Bentley H, Holm RH, Smith T. Generative AI as a tool for environmental health research translation. GeoHealth 2023;7:1–4. 10.1029/2023GH000875 PMC10369501 37502196

[4] Barros A , Prasad A, Śliwa M. Generative artificial intelligence and academia: implication for research, teaching and service. Manag Learn 2023;54:597–604.

[5] Khalifa M , Albadawy M. Using artificial intelligence in academic writing and research: an essential productivity tool. Comput Methods Prog Biomed Update 2024;5:100145.

[6] Stokel-Walker C , Van Noorden R. What ChatGPT and Generative AI mean for science. Nature 2023;614:214–216. 36747115 10.1038/d41586-023-00340-6

[7] Dashti M , Londono J, Ghasemi S, Moghaddasi N. How much can we rely on artificial intelligence chatbots such as the ChatGPT software program to assist with scientific writing? J Prosthet Dent 2025;133:1082–1088. 37438164 10.1016/j.prosdent.2023.05.023

[8] Harvard University. Research With Generative AI. 2024. https://www.harvard.edu/ai/research-resources (23 February 2024, date last accessed).

[9] Flanagin A , Bibbins-Domingo K, Berkwits M, Christiansen SL. Nonhuman ‘authors’ and implications for the integrity of scientific publication and medical knowledge. J Am Med Assoc 2023;329:637–639. 10.1001/jama.2023.1344 36719674

[10] Elsevier. *The Use of Generative AI and AI-Assisted Technologies in Scientific Writing*. 2024. https://www.elsevier.com/about/policies-and-standards/generative-ai-policies-for-journals (23 February 2024, date last accessed).

[11] Cardon P , Fleischmann C, Aritz J, Logemann M, Heidewald J. The challenges and opportunities of AI-assisted writing: developing AI literacy for the AI age. Bus Prof Commun Q 2023;86:257–295.

[12] Microsoft. Copilot (Feb 24 version) Response to Reviewer Data Collection Form. 2024. https://copilot.microsoft.com (12 February 2024, date last accessed).

[13] OpenAI. *ChatGPT (Feb 24 version) Response to Reviewer Data Collection Form*. 2024. https://chatgpt.com/ (12 February 2024, date last accessed).

[14] Google. *Gemini 1.0 (Feb 24 version) Response to Reviewer Data Collection Form*. 2024. https://gemini.google.com (12 February 2024, date last accessed).

[15] Samuel A. *How to Build Your Own AI Assistant: Harvard Business Review*. 2025. https://hbr.org/2025/03/how-to-build-your-own-ai-assistant.

