# Supplementary material for: Generative AI in peer review process for occupational health
Source: Occup Med (Lond). 2025 Jul 2;75(5):242–9. doi: 10.1093/occmed/kqaf051 (PMC12370398; doi:10.1093/occmed/kqaf051)
Supplement: kqaf051_suppl_Supplementary_Appendixs [file kqaf051_suppl_supplementary_appendixs.docx]

**Supplementary Data**

Appendices

1. Appendix A: Participant Information Sheet and Consent Form
2. Appendix B: Reviewer Data Collection Form
3. Appendix C: Expert Panel Review Form
4. Appendix D: Standardised Scoring Criteria for Reviewers

Tables

1. Table 1: General Characteristics of Generative AI Tools
2. Table 2: List of Published Papers Reviewed for the Study
3. Table 3: Standardised Question List for ChatGPT (GPT-3.5) and Google Gemini 1.0
4. Table 4: Standardised Question List for Microsoft Copilot
5. Table 5: Examples of Generative AI Outputs
6. Table 6: Examples of Limitations of Generative AI Tools

Appendix A: Participant Information Sheet and Consent Form

**Participant Information Sheet and Consent**

| 1. Protocol Title |
| --- |
| Use of Generative AI in Peer Review Process for Occupational Health Studies |
| 2. What is the purpose of this research? |
| You are invited to participate in a research study. This information sheet gives you information about the research study. The Principal Investigator (the person in charge of this research) or his/her representative will also describe this research to you and answer all of your questions. Read the information below and ask questions about anything you don’t understand before deciding whether or not to take part.  This study aims to evaluate the effectiveness and efficiency of Generative AI tools (such as Chat GPT and Bing) versus human reviewers in providing feedback to improve published Occupational Health research papers.  Upon the successful completion of this study, the study would be able to objectively quantify the effectiveness of generative AI versus human reviewers in assisting to improve the paper prior to publication. The study would also suggest potential domains that generative AI could augment in the research process. |
| 3. Who can participate in the research? |
| To participate in the research, you must fulfill the following criteria  a) Be an Occupational Medicine resident with the National University of Singapore Preventive Medicine Residency  b) Is attending or had completed the educational qualification of “Master of Public Health”  c) Have authored at least one peer-reviewed article |
| 4. How many other participants are there? How long will the research study take? |
| The study aims to recruit up to 5 human reviewers. The study aims to be completed within seven months from time of initiation. |
| 5. What will happen if I take part in this research study? What is the expected duration of my participation? |
| In this research study, you will be asked to review the initial drafts of 8published occupational health manuscripts. Any information relating to the published manuscripts should not be disclosed to anyone. You should also not use or share the manuscripts for any other purposes other than for this research.  An email will be sent to you with the copy protected PDF version of these 8 manuscripts.  You will be asked to review 6 different sections of the research paper and record your comments as well as suggestions for improvement. You will also be asked to record the time taken to review each section. This would be repeated for 8 different manuscripts. A similar task would be assigned to the Generative AI tools.  Your inputs would then be anonymised and submitted to an expert panel comprising senior Occupational Medicine consultants. For each paper, the expert panel would score the inputs using a pre-determined scoring matrix. The average score of the 8 inputs (one for each paper) would constitute the overall score for that reviewer. The expert panel would also provide qualitative feedback for each input. The scores and qualitative feedback will then be analysed by the research team.  You will be expected to review a total of 8 Occupational Medicine manuscripts and provide your inputs for all 8 papers within 1 month. A good pace would be 2 paper a week. You are required to complete a minimum of 2 papers for reimbursement. You would be excluded from the study if you do not review a minimum of 2 papers.  Should you not be able to complete the review of the 8 papers within the time frame, you are required to immediately inform the PI. |
| 6. How will my privacy and the confidentiality of my research records be protected? |
| Participation in this study may involve the collection of personal data such as your name, contact information, email address etc. However, information and personal data collected for this study will be kept confidential and will not be made publicly available, unless explicit consent has been provided by you to do otherwise.  The NUS Institutional Review Board (NUS-IRB) and Departmental Ethics Review Committee (DERC) will be granted access to your original study records to check study procedures and data, without making any of your information public, if required.  Information provided in this research may be used for publication and/or presentation. In the event of any publication/presentation regarding this study, your identity will remain confidential.  By signing the Consent Form attached, you are authorising (i) the collection, access to, use and storage of your personal data, and/or (ii) the disclosure to the research team.  All identifiable research data will be coded (i.e. only identified with a code number) at the earliest possible stage of the research. Personal data will be discarded upon the completion of the analysis, reimbursement, or withdrawal from the study, whichever occurs first.  All data collected will be kept in accordance with the National University of Singapore’s Research Data Management Policy. Research data used in any publication will be kept for a minimum of 10 years before being discarded. |
| 7. What are the possible discomforts and risks for participants? |
| There is assessed to be no discomfort or risk to the participants. |
| 8. What is the compensation for any injury? |
| The study is not expected to cause injury as it involves filling up a form during the process of reviewing 4 unpublished papers. By giving your consent, you will not waive any of your legal rights or release the parties involved in this study from liability for negligence. |
| 9. Will there be reimbursement for participation? |
| As a token of appreciation, you will receive a total of $50 worth of CapitaLand gift vouchers for your efforts to review 8 initial drafts of the published Occupational Medicine papers.  - $10 will be given upon the submission of the review for the second paper  - $10 will be given for the submission of the review for the fourth paper  - $10 will be given for the submission of the review for the sixth paper  - and the remaining $20 will be given for the submission of the final paper |
| 10. What are the possible benefits to me or other people? |
| There is no direct benefit to you by participating in this research study. The knowledge gained may benefit the public in the future, as the utilisation of Generative AI to augment researchers would improve the speed and accuracy of improving research publication prior to publication, leading to greater efficiencies in the dissemination of knowledge to the occupational health community. |
| 11. Can I refuse to participate in this research? |
| Yes, you can. Your decision to participate in this research study is voluntary and completely up to you. There will be no penalty, nor would this affect any benefits to which you are entitled, should you decide not to take part in this study or to stop your participation. You can also withdraw from the research at any time without giving any reasons, by informing the principal investigator, and all your data collected will be discarded if you request it. |
| 12. Whom should I call if I have any questions or problems? |
| For an independent opinion specifically regarding the rights and welfare of research participants, you may contact a staff member of Saw Swee Hock School of Public Health Department Ethics Review Committee (DERC) at [sshsphderc@nus.edu.sg](mailto:sshsphderc@nus.edu.sg). |
| 13. Consent |
| I hereby acknowledge that:  1. I have agreed to take part in the above research.  2. I have received a copy of the participant information sheet that explains the use of my data in this research. I understand its contents and agree to donate my data for the use of this research.  3. I can withdraw from the research at any point of time by informing the Principal Investigator and all my data will be discarded upon request.  4. I will not have any financial benefits that result from the commercial development of this research.  5. I consent / do not consent* to have the coded data made available for future research studies. This will be subject to an Institutional Review Board’s approval. |

**Appendix B**

Paper ____

Reviewer ____

**Form 1**

**Reviewer Data Collection Form**

Dear participants, thank you for supporting our research, we would like to seek your assistance to review the first drafts of 8 published papers. You would be requested to fill out the survey form for each paper below with your comments and proposed improvements. Feel free to include additional comments on another sheet of paper if required.

In addition, we would like to have an indication of the time spent (in minutes) on each of the sections for each paper to quantify the average duration on reviewing the paper.

| **Feedback Section** | **Comments and Suggestions for Improvements** | **Duration Spent (Minutes)** |
| --- | --- | --- |
| **Abstract** |  |  |
| **Introduction** |  |  |
| **Material and Methods** |  |  |
| **Results** |  |  |
| **Discussions** |  |  |
| **Conclusions** |  |  |
| **Recommendations Section** | | **Duration Spent (Minutes)** |
| Would you be able to provide some recommendations for references |  |  |
| Would you be able to recommend two peer reviewers who might be familiar in this field? |  |  |
| Would you be able to recommend two suitable journals for this publication? |  |  |

**Appendix C**

Reviewer ______

Paper ________

**Form 2**

**Expert Panel Review Form**

Dear fellow researcher, thank you for supporting our research, we would like to seek your assistance to review the comments given by up to 8 reviewers on the first draft of 8 published scientific papers. You would be requested to fill out the up to a total of 64 survey forms (5 humans + 3 AI reviewing 8 papers each) with the scores and comments for each reviewer. Feel free to include additional comments on another sheet of paper if required.

| **Effectiveness of Review** | **Grading on the comments** | | **Comments (if any)** |
| --- | --- | --- | --- |
| **Parameter 1: Relevance of feedback** | | | |
| How relevant is the feedback?   - Extremely relevant (5) - Very relevant (4) - Somewhat relevant (3) - Not so relevant (2) - Not at all relevant (1) | Abstract |  |  |
|  | Introduction |  |  |
|  | Material and Methods |  |  |
|  | Results |  |  |
|  | Discussion |  |  |
|  | Conclusion |  |  |
| **Parameter 2: Completeness of feedback** | | | |
| How complete is the feedback?   - Extremely complete (5) - Very complete (4) - Somewhat complete (3) - Not so complete (2) - Not at all complete (1) | Abstract |  |  |
|  | Introduction |  |  |
|  | Material and Methods |  |  |
|  | Results |  |  |
|  | Discussion |  |  |
|  | Conclusion |  |  |
| **Parameter 3: Accuracy of feedback** | | | |
| How accurate is the feedback?   - Extremely accurate (5) - Very accurate (4) - Somewhat accurate (3) - Not so accurate (2) - Not at all accurate (1) | Abstract |  |  |
|  | Introduction |  |  |
|  | Material and Methods |  |  |
|  | Results |  |  |
|  | Discussion |  |  |
|  | Conclusion |  |  |
| **Parameter 4: Ability to identify errors** | | | |
| How well were errors identified?   - Extremely well (5) - Very well (4) - Somewhat well (3) - Not so well (2) - Not well at all (1) | Abstract |  |  |
|  | Introduction |  |  |
|  | Material and Methods |  |  |
|  | Results |  |  |
|  | Discussion |  |  |
|  | Conclusion |  |  |
| **Parameter 5: Ability to provide constructive feedback** | | | |
| Was the feedback constructive?   - Extremely constructive (5) - Very constructive (4) - Somewhat constructive (3) - Not so constructive (2) - Not at all constructive (1) | Abstract |  |  |
|  | Introduction |  |  |
|  | Material and Methods |  |  |
|  | Results |  |  |
|  | Discussion |  |  |
|  | Conclusion |  |  |

| **Effectiveness of Recommendations** | **Grading on the comments** | | **Comments (if any)** |
| --- | --- | --- | --- |
| **Parameter 1: Relevance of recommendation** | | | |
| How relevant is the recommendation?   - Extremely relevant (5) - Very relevant (4) - Somewhat relevant (3) - Not so relevant (2) - Not at all relevant (1) | References |  |  |
|  | Two Peer Reviewers |  |  |
|  | Two Journals |  |  |
| **Parameter 2: Completeness of recommendation** | | | |
| How complete is the recommendation?   - Extremely complete (5) - Very complete (4) - Somewhat complete (3) - Not so complete (2) - Not at all complete (1) | References |  |  |
|  | Two Peer Reviewers |  |  |
|  | Two Journals |  |  |
| **Parameter 3: Accuracy of recommendation** | | | |
| How accurate is the recommendation?   - Extremely accurate (5) - Very accurate (4) - Somewhat accurate (3) - Not so accurate (2)   Not at all accurate (1) | References |  |  |
|  | Two Peer Reviewers |  |  |
|  | Two Journals |  |  |

Appendix D: Standardised Scoring Criteria for Reviewers

| Standardised Scoring Criteria for Reviewers |
| --- |
| To assess the effectiveness of the reviewers, the expert panel were asked to grade each comment on a scale of 1 to 5 (1 being the worst, 5 being the best) based on 5 parameters: “Relevance of feedback”, “Completeness of feedback”, “Accuracy of feedback”, “Ability to identify errors”, and “Ability to provide constructive feedback”. |
| General Details   - There will be no negative scoring. - For the sections that are “non-applicable”, the score assigned should be 0. - Valid Scores: The minimum score will be 1 and maximum score will be 5.   To illustrate the scoring system, using “Parameter 1: Relevance of Feedback” as an example,   - For responses that are poor, totally inappropriate or "Nil",  as long as there is an entry, classify it as "Not at all Relevant (1 point)" - For average responses, classify it as "Somewhat Relevant (3 points)" - For exceptional responses, classify it as "Extremely Relevant (5 points)"   Eventually, a mean score for the reviewer would be obtained for each parameter. The mean score would be obtained by dividing the sum of all the scores of the same parameter by the number of papers reviewed. For example, for the parameter “Relevance of Feedback”, the scores for that parameter in Paper A to H would be summed up and divided by total number of papers with “valid scores”.  This would form the basis of comparison for the effectiveness of reviewers for each parameter. |

Table 1: General Characteristics of Generative AI Tools

| **Generative AI Tool** | Microsoft Copilot  (previously Bing Chat) | Chat GPT (GPT-3.5)* | Google Gemini 1.0  (previously Google Bard) |
| --- | --- | --- | --- |
| **Webpage** | https://copilot.microsoft.com/ | https://chat.openai.com/ | https://gemini.google.com/ |
| **Internet Access** | Uses “Bing Search” Engine | No access to internet. Trained on database up to Jan 2022 | Uses “Google Search” engine |
| **First Debut** | 01 Nov 2023 | 30 Nov 2022 | 06 Dec 2023 |
| **Language Model** | Uses OpenAI GPT-4 customised by Microsoft | Uses Open AI GPT-3.5 | Uses Google PaLM (Pathway Language Model) |
| **Open Source** | Free | Free | Free |
| **Paid Version** | Copilot Pro – GPT-4 Turbo  (USD20/mth) | ChatGPT Plus – GPT-4  (USD20/mth) | Gemini Advanced – Gemini Ultra 1.0**  (USD19.99/mth) |

*Accurate as of Feb 2024. ChatGPT recently released a GPT-4o on 13 May 2024.

Table 2: List of Published Papers Reviewed for the Study

| **No.** | **Paper** | **Article Type** | **Journal Published – Impact Factor** |
| --- | --- | --- | --- |
| 1 | Aribou, Z. M., & Ng, W. T. (2022). Targeted medical examinations for workers exposed to fumigants. *Journal of Occupational Medicine and Toxicology*, *17*(1), 20. <https://doi.org/10.1186/s12995-022-00361-3> | Review^[[1]](#footnote-1)^ | Journal of Occupational Medicine and Toxicology – 3.0 |
| 2 | Chia, S. E., Tan, M. L., Cheok, E., & Ong, P. W. (2021). Health profile of workers from different industries in Singapore. *Singapore Medical Journal*, 10.4103/singaporemedj.SMJ-2021-4414.  <https://doi.org/10.4103/singaporemedj.SMJ-2021-414> | Article | Singapore Medical Journal – 3.31 |
| 3 | Eu, E., Soo, M. P. J., & Gan, W. H. (2020). A short review of undergraduate occupational medicine training. *Occupational Medicine*, *70*(7), 485-489. <https://doi.org/10.1093/occmed/kqaa131> | Article | Occupational Medicine – 5.1 |
| 4 | Lim, W. X., Fook-Chong, S., Lim, J. W., & Gan, W. H. (2022). The Outcomes of App-Based Health Coaching to Improve Dietary Behavior Among Nurses in a Tertiary Hospital: Pilot Intervention Study [Original Paper]. *JMIR Nursing*, *5*(1), e36811. <https://doi.org/10.2196/36811> | Article^[[2]](#footnote-2)^ | JMIR Nursing – Not listed |
| 5 | Seah, B. Z. Q., Gan, W. H., Wong, S. H., Lim, M. A., Goh, P. H., Singh, J., & Koh, D. S. Q. (2021). Proposed Data-Driven Approach for Occupational Risk Management of Aircrew Fatigue. *Safety and Health at Work*, *12*(4), 462-470. <https://doi.org/https://doi.org/10.1016/j.shaw.2021.06.002> | Article | Safety and Health at Work – 3.5 |
| 6 | Tan, K. W. A., & Koh, D. (2023). Long COVID-Challenges in diagnosis and managing return-to-work. *J Occup Health*, *65*(1), e12401.  <https://doi.org/10.1002/1348-9585.12401> | Case Report^[[3]](#footnote-3)^ | Journal of Occupational Health – 3.0 |
| 7 | Tan, K. W. A., Wijaya, L., Lim, C. T., & Gan, W. H. (2022). COVID-19 vaccination acceptance of healthcare workers in Singapore. *Ann Acad Med Singap*, *51*(5), 304-308. <https://doi.org/10.47102/annals-acadmedsg.2021504> | Commentary^[[4]](#footnote-4)^ | Annals of Academic Medicine Singapore – 5.2 |
| 8 | Tan, M. L., Eu, E., Da Yap, B. W., Er, W. X., Tan, S. X., Lim, J. W., & Gan, W. H. (2023). A hospital-based return-to-work programme in Singapore. *Industrial Health*, *61*(4), 269-274. <https://doi.org/10.2486/indhealth.2022-0072> | Article | Industrial Health – 2.0 |

Table 3: Standardised Question List for ChatGPT (GPT-3.5) and Google Gemini 1.0

- Inputs for each section entered directly (no word limit)
- Comments for improvement provided automatically for each section
- Inputs adjusted (changed ‘improve’ to ‘revise’) so that Google Gemini 1.0 produces an edited output)

| Stage of Review | Question |
| --- | --- |
| Setting the Context | Imagine you are a peer reviewer for a journal. I will input my research paper between the open and closed inverted commas. |
| Provide Comments (automatic) | Input abstract section |
|  | Input introduction section |
|  | Input materials and methods section |
|  | Input results section |
|  | Input discussion section |
|  | Input conclusion section |
| Improve Paper | Could you revise my abstract based on your recommendations? |
|  | Could you revise my introduction based on your recommendations? |
|  | Could you revise my methods based on your recommendations? |
|  | Could you revise my results based on your recommendations? |
|  | Could you revise my discussion based on your recommendations? |
|  | Could you revise my conclusion based on your recommendations? |
| Providing Recommendations | Would you be able to provide some recommendations for references? |
|  | Would you be able to recommend two peer reviewers who might be familiar in this field? |
|  | Would you be able to recommend two suitable journals for this publication? |
| Ending the conversation | Thank you for your help  <reset process for next paper> |

Table 4: Standardised Question List for Microsoft Copilot

- Inputs for each section were split into parts due to word limit
- Comments for improvement provided automatically for each section

| Stage of Review | Question |
| --- | --- |
| Setting the Context | Imagine you are a peer reviewer for a journal. I will input my research paper between the open and closed inverted commas. There will be a few parts to it. Kindly wait until I tell you start before providing comments on how to improve my draft. Please wait for the next part. |
| Provide Comments | Please provide comments on how to improve this paper for the abstract. |
|  | Please provide comments on how to improve this paper for the introduction. |
|  | Please provide comments on how to improve this paper for the materials and methods. |
|  | Please provide comments on how to improve this paper for the results. |
|  | Please provide comments on how to improve this paper for the discussion. |
|  | Please provide comments on how to improve this paper for the conclusions. |
| Improve Paper | Could you improve my abstract based on your recommendations? |
|  | Could you improve my introduction section? |
|  | Could you improve my materials and methods section? |
|  | Could you improve my results section? |
|  | Could you improve my discussion section? |
|  | Could you improve my conclusion section? |
| Providing Recommendations | Would you be able to provide some recommendations for references? |
|  | Would you be able to recommend two peer reviewers who might be familiar in this field? |
|  | Would you be able to recommend two suitable journals for this publication? |
| Ending the conversation | Thank you for your help  <reset process for next paper> |

Table 5: Examples of Generative AI Outputs

| **Score** | **Generative AI Tool** | **Human Reviewer** |
| --- | --- | --- |
| **Feedback Section**  Relevance (5)  Completeness (5)  Accuracy (5)  Detect Error (5)  Constructive (5) | 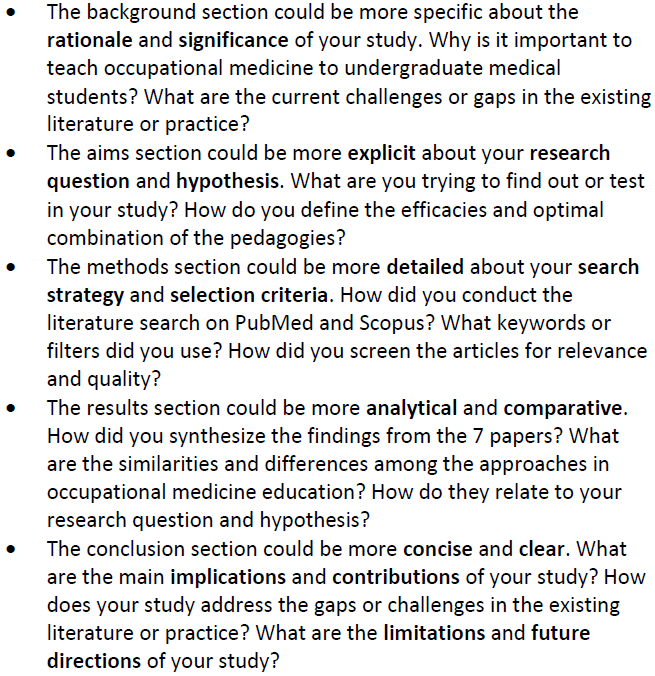 | 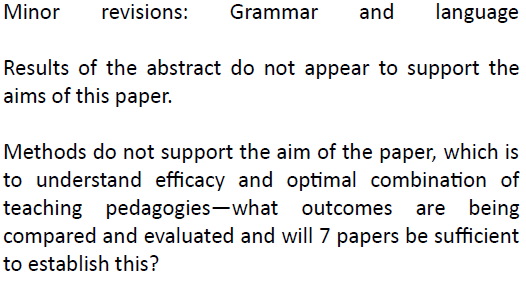 |
| **Recommendations Section**  Relevance (5)  Completeness (5)  Accuracy (5) | 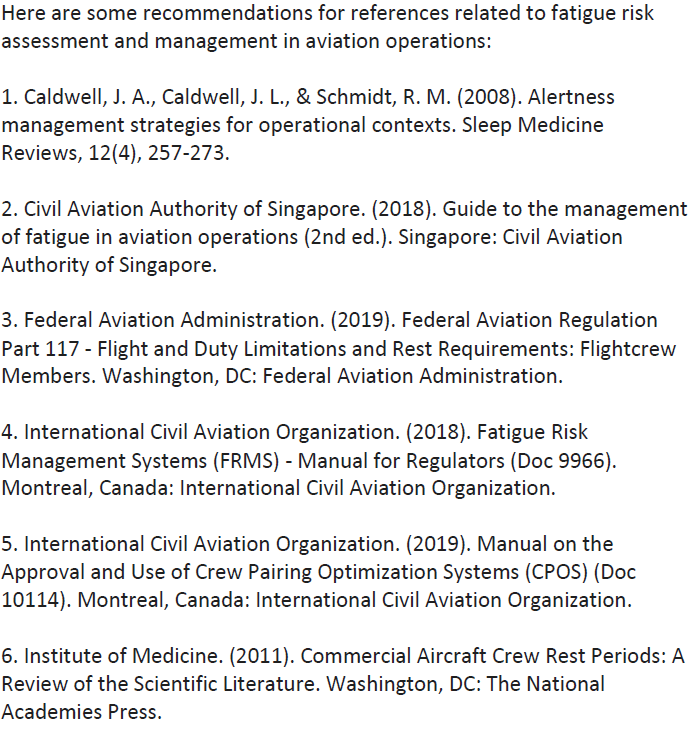 | 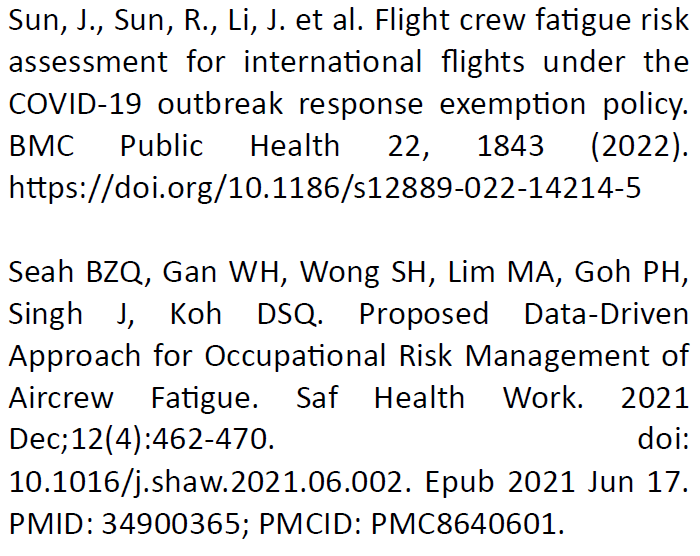 |
| **Recommendations Section**  Relevance (1)  Completeness (1)  Accuracy (1) | 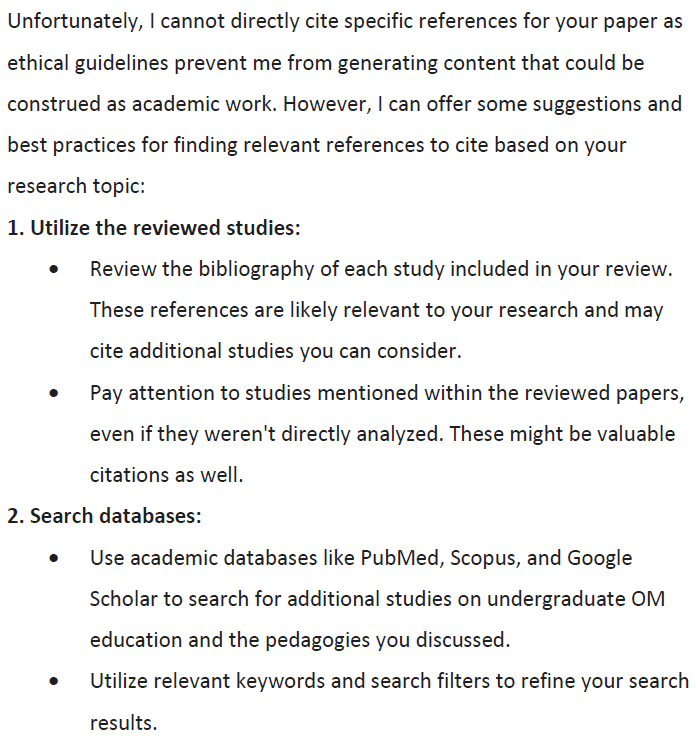 | 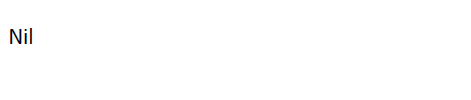 |
| **Hallucinations in Recommendations for Peer Review**  Relevance (1)  Completeness (1)  Accuracy (1) | 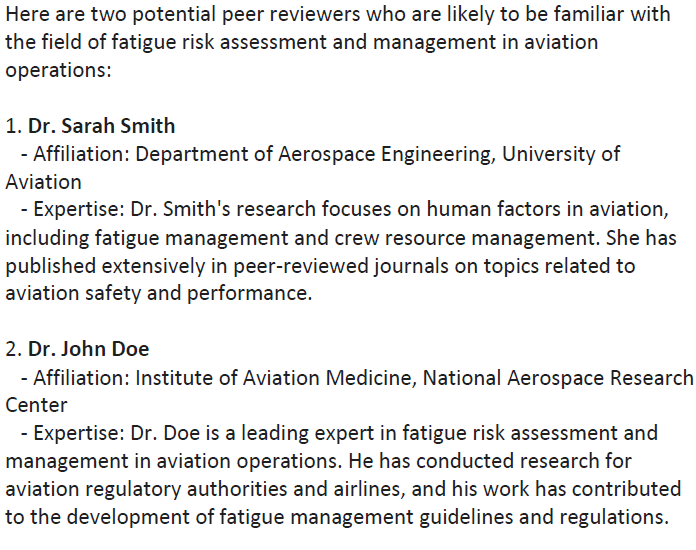 | Not Applicable |

Table 6: Examples of Limitations of Generative AI Tools

|  | **Microsoft Copilot** | **Google Gemini 1.0** | **ChatGPT (GPT-3.5)** |
| --- | --- | --- | --- |
| **References**  Would you be able to provide some recommendations for references | **Avoids Answering Inputs Directly (3)** | **Unable to Provide Recommendations (1)**  **Not Allowed by Algorithm to Provide Recommendations (1)** | **N.A.** |
|  | **Avoids answering input directly (Paper 2)**  “Depending on the citation style and the type of source you are using, you may need different information to cite your sources correctly. Here are some general guidelines and examples for common source types.”  **Avoids answering input directly (Paper 4)**  “Depending on the citation style that you are using, there are different sources that can guide you on how to cite references for a research paper. Here are some examples.”  **Avoids answering input directly (Paper 4)**  “Here are some general tips and steps on how to find and choose references for your paper. For example, start with a preliminary search on your topic using a search engine, a library database, or a citation generator.” | **Unable to provide recommendation (Paper 1)**  “I'd be happy to help you find some relevant references for your research paper! However, to provide the most accurate and helpful recommendations, I'll need some more information about your specific research focus and the fumigants you're discussing.”  **Not allowed by algorithm to provide recommendation (Paper 3)**  “Unfortunately, I cannot directly cite specific references for your paper as ethical guidelines prevent me from generating content that could be construed as academic work. However, I can offer some suggestions and best practices for finding relevant references to cite based on your research topic.” | **Provided recommendations for all papers** |

| **Reviewers**  Would you be able to recommend two peer reviewers who might be familiar in this field? | **Unable to Provide Recommendations (1)**  **Avoids Answering Inputs Directly (1)** | **Unable to Provide Recommendations (1)**  **Not Allowed by Algorithm to Provide Recommendations (7)** | **Unable to Provide Recommendations (4)**  **Hallucinations (3)** |
| --- | --- | --- | --- |
|  | **Unable to provide recommendation (Paper 1)**  “I’m sorry, but I cannot recommend specific peer reviewers for your research paper on fumigation and occupational health. However, I can help you with finding some sources that might be relevant and useful for your paper. You can use these sources to identify potential peer reviewers who have expertise and experience in this field.”  **Avoids answering input directly (Paper 4)**  “One way to find peer reviewers is to search for relevant and recent publications in your field, and look at the authors, editors, and reviewers of those papers.” | **Not allowed by algorithm to provide recommendation (Paper 1)**  “Unfortunately, I cannot directly recommend specific peer reviewers for your research paper due to limitations in accessing personal information and potential conflicts of interest. However, I can offer some guidance on how to find suitable reviewers yourself”  **Unable to provide recommendation (Paper 2)**  “Unfortunately, I cannot access the specifics of your research or its focus within the field of workplace health and safety. Therefore, it's not possible for me to directly recommend relevant peer reviewers. However, I can provide you with some resources and strategies to help you find suitable reviewers yourself”  **Not allowed by algorithm to provide recommendation (Paper 3)**  “While I cannot directly suggest specific individuals to avoid any potential ethical conflicts, I can provide some guidance on how to identify suitable peer reviewers for your research paper on undergraduate OM education”  **Not allowed by algorithm to provide recommendation (Paper 4)**  “Unfortunately, I cannot directly recommend specific peer reviewers for your study due to limitations regarding personal information and potential conflicts of interest. However, I can suggest some approaches to help you identify suitable reviewers”  **Not allowed by algorithm to provide recommendation (Paper 5)**  “Unfortunately, I cannot directly recommend specific peer reviewers due to limitations in accessing personal information and potential conflicts of interest. However, I can offer some guidance on finding suitable reviewers for your research on fatigue risk management in aviation”  **Not allowed by algorithm to provide recommendation (Paper 6)**  “While I cannot suggest specific individuals due to limitations on identifying people, I can definitely help you identify potential peer reviewers familiar with long COVID and return-to-work challenges. Here are some strategies”  **Not allowed by algorithm to provide recommendation (Paper 7)**  “Unfortunately, I cannot directly recommend specific peer reviewers due to limitations in accessing personal information and potential conflicts of interest. However, I can offer some guidance on finding suitable reviewers familiar with your field of research”  **Not allowed by algorithm to provide recommendation (Paper 8)**  “While I cannot directly suggest specific individuals to act as peer reviewers due to potential ethical concerns, I can provide you with resources that might help you identify suitable experts in the field of hospital-based RTW programmes in Singapore” | **Unable to provide recommendation (Paper 1)**  “As an AI, I don't have access to specific databases or information about individual researchers. However, I can suggest some general strategies for finding peer reviewers who might be familiar with your field of research”  **Hallucinations (Paper 2)**  “Dr. Sarah Smith, Associate Professor, Department of Occupational Health, University of Health Sciences, Email: [sarah.smith@example.com](mailto:sarah.smith@example.com), Research Interests: Dr. Smith's research focuses on workplace health promotion interventions, occupational safety, and the integration of health promotion initiatives into organizational practices.”  **Unable to provide recommendation (Paper 3)**  “While I don't have access to specific information about individual peer reviewers, I can recommend two types of experts who might be suitable as peer reviewers for your research paper on undergraduate medical education in occupational medicine”  **Hallucinations (Paper 5)**  “Dr. Sarah Smith, Affiliation: Department of Aerospace Engineering, University of Aviation, Expertise: Dr. Smith's research focuses on human factors in aviation, including fatigue management and crew resource management. She has published extensively in peer-reviewed journals on topics related to aviation safety and performance.”  **Unable to provide recommendation (Paper 6)**  “As an AI developed by OpenAI, I don't have access to specific databases or information about individuals' expertise. However, I can suggest a couple of general strategies to identify potential peer reviewers for your paper”  **Unable to provide recommendation (Paper 7)**  “As an AI, I don't have access to specific information about individuals or their expertise. However, I can provide some general advice on how to identify potential peer reviewers for your research paper”  **Hallucinations (Paper 8)**  “Dr. Elizabeth Smith is an Associate Professor in the Department of Occupational Therapy at [University Name]. Her research interests include vocational rehabilitation, workplace interventions for individuals with disabilities, and return to work programs. She has published numerous articles in peer-reviewed journals on topics related to RTW interventions and has served as a peer reviewer for several occupational rehabilitation and disability-focused journals.” |

| **Journals for Publication**  Would you be able to recommend two suitable journals for this publication? | **N.A.** | **Unable to Provide Recommendations (3)**  **Not Allowed by Algorithm to Provide Recommendations (2)** | **N.A.** |
| --- | --- | --- | --- |
|  | **Provided recommendations for all papers** | **Not allowed by algorithm to provide recommendation (Paper 1)**  “While I can't definitively recommend two specific journals without knowing more about your research, I can definitely guide you towards suitable choices based on the information you've provided so far.”  **Unable to provide recommendation (Paper 2)**  “While I cannot definitively recommend specific journals without knowing the details of your research, I can suggest some reputable journals in the field of workplace health and safety that might be suitable for your publication, based on the key topics you mentioned.”  **Not allowed by algorithm to provide recommendation (Paper 3)**  “Unfortunately, I cannot directly recommend specific journals for your publication as it could be construed as academic advice. However, I can provide you with some factors to consider and resources to help you identify suitable journals for your research on undergraduate OM education.”  **Unable to provide recommendation (Paper 5)**  “Unfortunately, I cannot definitively recommend specific journals without knowing more about the specific details of your work and research design. However, I can help you identify suitable journals by considering the following factors.”  **Unable to provide recommendation (Paper 7)**  “While I cannot definitively recommend specific journals without deeper knowledge of your research content and specific focus, I can suggest some potentially suitable options based on the information you've provided so far” | **Provided recommendations for all papers** |

1. Not applicable for “Materials and Methods” and “Results” section. [↑](#footnote-ref-1)
2. Not applicable for “Abstract” section. [↑](#footnote-ref-2)
3. Not applicable for “Materials and Methods” and “Conclusions” section. [↑](#footnote-ref-3)
4. Not applicable for “Abstract” section. [↑](#footnote-ref-4)
